# Preparation of monoclonal antibodies against gamma-type phospholipase A_2_ inhibitors and immunodetection of these proteins in snake blood

**DOI:** 10.1186/s40409-017-0128-5

**Published:** 2017-08-03

**Authors:** Jingjing Li, Ying Xiong, Shimin Sun, Lehan Yu, Chunhong Huang

**Affiliations:** 1 Department of Biochemistry, College of Basic Medical Science, Nanchang University, Nanchang, 330006 China; 2 Second Affiliated Hospital to Nanchang University, Nanchang University, Nanchang, 330006 China; 3 Jiangxi Province Key Laboratory of Tumor Pathogens and Molecular Pathology, Nanchang University, 461 Bayi Avenue, Nanchang, 330006 China

**Keywords:** Monoclonal antibody, Phospholipase A_2_ inhibitor, Epitope prediction

## Abstract

**Background:**

The gamma-type phospholipase A_2_ inhibitor (PLIγ) is a natural protein commonly found in snake serum, which can neutralize pathophysiological effects of snake venom phospholipases A_2_. Therefore, this protein is a potential candidate to the development of a novel antivenom. To the best of our knowledge, there is no antibody currently available for PLIγ identification and characterization.

**Methods:**

Bioinformatics prediction of epitope using DNAStar software was performed based on the sequence of *Sinonatrix annularis* PLIγ (SaPLIγ). The best epitope ^151^CPVLRLSNRTHEANRNDLIKVA^172^ was chosen and synthesized, and then conjugated to keyhole limpet hemocyanin and bovine serum albumin for use as an immunogen and plate-coating antigen, respectively.

**Results:**

Eighteen IgG anti-PLIγ mAb hybridoma cell strains were obtained, and all the mAbs had positive interaction with recombinant His6-PLIγ and natural SaPLIγ. Moreover, the mAb from 10E9 strain was also successfully used for the immunodetection of other snake serum PLIγs. cDNA sequence alignment of those PLIγs from different snake species showed that their epitope segments were highly homologous.

**Conclusions:**

The successful preparation of anti-PLIγmAb is significant for further investigation on the relationship between the structure and function of PLIγs, as well as the interaction between PLIγs and PLA_2_s.

## Background

Although appeals to improve the prevention and care of snakebites date back more than a century, they remain a neglected medical problem, leading annually to more than 125,000 deaths [1, 2]. Snake venom phospholipase A_2_s (svPLA_2_) play a critical role in the early morbidity and mortality due to snakebites, causing paralysis and death, as well as tissue destruction and disorders of homeostatic mechanisms [3–5]. PLA_2_s are commonly found in the venom of all snakes including the ones from the families Hydrophiidae, Viperidae, Elapidae and Crotalinae. However, it is rarely found in Colubridae family (non-venomous snakes), which indicates that PLA_2_s are vital toxic components of snake venoms [6].

Phospholipase A_2_ or PLA_2_ (E.C. 3.1.1.4) is a class of enzyme that hydrolyzes the sn-2-acyl groups on phospholipid molecules, releasing free fatty acids and lysophospholipids as products. SvPLA_2_s are known to belong to group I (Elapidae/Hydrophidae) and II (Viperidae/Crotalidae) of PLA_2_s [7]. However, unlike other mammalian PLA_2_s, svPLA_2_s have strong characteristics of neurotoxicity, myotoxicity, hemorrhage, cardiac toxicity, platelet aggregation, presynaptic and postsynaptic activities [5, 8, 9]. Victims of snakebite are also subjected to systematic inflammation response syndrome (SIRS) and local pain and necrosis, due to the activation of the arachidonic acid pathway and accumulation of prostaglandins and leukotrienes, a process induced by increasing PLA_2_ enzymatic activity [10]. In summary, svPLA_2_s are key toxic components of snake venom and, therefore, comprise a good target for antivenom development.

Clinically, antivenom is the current most effective medicine for clinical treatment of snakebites. However, the shortage of antivenom has been increasingly worrying physicians and scientists in recent years. Initially, a correct diagnosis of snakebite is experience dependent and critical for victims who can be treated with appropriate antivenoms. Second, the burden of snakebite is worldwide heavy, especially in Africa, Asia and Latin America [11–13]. The categories of antivenoms are far less than the number of venomous snake species. For example, there are about 60 venomous snakes in China, while there are only four antivenoms produced by the sole manufacturer in China (Shanghai Serum Biological Technology). Even worse, due to the low profitability, the French pharmaceutical company Sanofi Pasteur had ceased the production of Fav-Afrique, the most effective antivenom against African vipers, mambas and cobras. This scenario has been leading the rural Africa into a major snakebite crisis [11]. These alarming situations appeal to appropriate replacements or new antivenom drug candidates. Finally, the common side effects of antivenom are also unfavorable for clinical application, i.e., the high risk of anaphylaxis and serum reactions [14, 15]. There is an urgent need for an effective and less costly replacement drug that can be administered in medical limited areas.

Due to these deficiencies in current snakebite treatment, there has been increasing interest in the search for naturally occurring molecules, which are able to inhibit the main pathophysiological effects induced by svPLA_2_s. These antidotes are mainly obtained from plant extracts, marine organisms and animal blood and are well summarized in the literature [16–18]. Some of these compounds have been studied for several years, but the treatment of snakebite has not met the necessary efficiency and effectiveness. Nevertheless, endogenous resistant proteins of svPLA_2_ in snake blood are often found effective in mitigating venom toxicity. Snakes generally produce svPLA_2_ inhibitors (PLI) as an innate immunity product for survival of their own species [19]. According to the structural features, snake PLI can be divided into α, β and γ types [20]. PLI-α and PLI-β are mainly distributed in Viperidae, and their inhibitory activities are mainly against their venom type II sPLA_2_ [21–23]. PLI-γ is a relatively nonspecific inhibitor that can inhibit svPLA_2_ subtypes I and II, bee venom, type III PLA_2_ from lizard venom, and mammalian sPLA_2_ (IB and IIA). PLIγs are widely found in the Elapidae, Hydrophiidae, Boidae and Colubridae snake families [20, 24–28].

To date, although there are more than 20 snake blood PLIγs described [20], there is no antibody against PLIγs available. In our previous study, we identified a novel PLIγ from a Chinese endemic non-venomous snake *Sinonatrix annularis* by ion exchange chromatography [29]. The PLIγ showed good anti-hemorrhagic effects against venoms of *Deinagkistrodon acutus, Agkistrodon hylas* and *Naja atra* [28]. In order to discover more natural PLIγs, as well as to investigate the interaction of PLIγ with svPLA_2_s, we used bioinformatics tools to predict B cells epitopes of *S. annularis* PLIγ. The resulted mAb is applicable for PLIγ immunodetection of a wide range of snake species.

## Methods

### Materials

Freund’s complete and incomplete adjuvants, bovine serum albumin (BSA), keyhole limpet hemocyanin (KLH), HAT medium supplement and HT medium supplement were purchased from Sigma Aldrich (USA). Protein G resin and the peroxidase conjugated goat antimouse IgG (IgG-HRP) were purchased from TransGen Biotech (China). Other reagents were of chemical grade from Beijing Solarbio Science & Technology Co. Ltd. (China). Female Balb/c mice were purchased from the Animal Research Institute of Nanchang University (China). Animal manipulation was performed in compliance with institutional guidelines approved by the Nanchang University (Nanchang, China).

### Epitopes prediction

The amino acid sequence of the PLIγ protein of *S. annularis* was used for epitope prediction. The linear B-cell epitopes of PLIγ were analyzed using DNASTar® protein sequence and structure analysis software. The secondary structure of the PLIγ – including α-helixes, β-sheets, β-turns and random coils – was construed with Chou-Fasman and Garnier-Robson parameters of Protean 3D software. The surface probability, flexibility and antigenicity of PLIγ were analyzed using Emini, Karplus-Schulz and Jameson-Wolf algorithm. Potential B cell epitopes were selected based on the following parameters: the epitope region containing β-turn or random coil, and few α-helix and β-sheet; the epitope peptides should display good hydrophilicity, high accessibility, high flexibility and strong antigenicity.

### Preparation of immunogen

Following the prediction results, the best epitope sequence was chosen for further study. The peptide was synthesized by Qiang Yao Biotechnology Company (China). The synthetic peptide was called SHE.

KLH was coupled to sulfo-GMBS to prepare maleimide activated carrier proteins (mcKLH). In brief, KLH and sulfo-GMBS (10 mg/mL) were mixed at 5:1 mass ratio and shaken at room temperature for 30 min, followed by 5 min of centrifugation at 12,000 rpm. The resulted supernatant was applied on a Sephadex G25 column to remove excess sulfo-GMBS. The mcKLH eluate was added dropwise into SHE peptide solution (6 mg/mL) and incubated at room temperature for 3 h. The coupling process was monitored using DTNB assay (Ellman’s reagent) and stopped when OD412 value decreased 2. The conjugated product was named SHE-KLH.

Bovine serum albumin (BSA) was used to prepare the coating antigen (SHE-BSA) using the same protocol as described above.

### mAb preparation

#### Mouse immunization

Four six-week-old BALB/c female mice were subcutaneously injected with 60 μg of SHE-KLH conjugate emulsified with Freund’s complete adjuvant as the primary immunization. Subsequently, 30 μg of SHE-KLH conjugate emulsified by Freund’s incomplete adjuvant was injected subcutaneously on days 14, 28, and 42, respectively, for a total of four immunizations. Seven days after the last immunization, serum from each mouse was verified for ability to bind SHE-BSA by indirect ELISA (iELISA). The mouse with the highest antibody titer was injected intraperitoneally with 50 μg of SHE-KLH conjugate and challenged with booster immunization. Three days later, the spleen was removed for hybridoma cell fusion.

#### Indirect ELISA

iELISA was used to assess epitope specific antibody titers of mouse sera or ascites fluid. SHE-BSA diluted with bicarbonate buffer (pH 9.6) (2 μg/mL) was added to 96-well microtiter plates at 100 μL/well and incubated at 4 °C overnight. The plate was washed three times with 300 μL/well of PBST (0.05% Tween-20 in 0.01 M PBS) and then blocked using 2% skim milk blocking buffer (200 μL/well) for 2 h at 37 °C followed by three washes. The immunized mouse serum was diluted 200 times in assay buffer, added into each well (100 μL/well) in a series of two-fold dilutions, and incubated at 37 °C for 1 h. The plate was then washed three times and subsequently incubated with goat anti-mouse IgG/HRP (1:20,000 dilutions in PBS) for 1 h at 37 °C (100 μL/well). After washing, coloring solution (100 μL/well) was added and incubated in dark for 5 min. The reaction was terminated by adding 50 μL/well of 2 mol/L H_2_SO_4_. The absorbance values were measured at two wavelengths (450, 630 nm) by an automatic ELISA plate reader.

#### Cell fusion and subtype analysis

The mouse with the highest antibody titers was selected from four mice, and was injected with 50 g of immunogen SHE-KLH intraperitoneally to obtain higher titers of antisera. The mouse was sacrificed through cervical vertebra dislocation, then soaked in 75% alcohol for 5 min. Splenocytes isolated from the immunized mouse and murine SP2/0 myeloma cells were mixed with PEG1500 and cultured in IMDM complete medium (with 15% serum) containing HAT and plated at 37 °C with 5% CO_2_. On the fifth day of fusion, the complete medium containing HT was used. Eight days later, culture supernatants and hybridoma screening were performed by iELISA. The antibody isotypes were determined using a Mouse Immunoglobulin Panel kit (Southern Biotech. USA) following the manufacturer’s instructions. The hybridoma cell line producing the highest binding capacity of immunoglobulins was injected intraperitoneally into mice to produce a higher amount of monoclonal antibody.

### Preparation of mAb

Ascites fluid was collected 7 to 10 days after injection of hybridoma cells. The ascites fluid was diluted 1:3 with equilibration buffer (20 mmol/L PB, 0.15 mmol/L KCl, pH 7.0) and centrifuged at 10,000 rpm for 20 min. The supernatant was further filtered through a 0.22-μm membrane to remove fat, cell debris and small particles. Ascites mAb was purified on a protein G chromatography column, which was pre-exhilarated by equilibration buffer and eluted by five times of bed volume elution buffer (0.1 mol/L glycine, pH 3.0) at a flow rate of 0.6 mL/min. The eluted mAb was immediately neutralized with an alkaline buffer (1 mol/L Tris-HCl, pH 9.0). The protein concentration and purity was determined following BCA assay kit and SDS-PAGE, respectively.

### Western blot analysis

Recombinant SaPLIγ was expressed in 6-histidine tag form by pET28c vector in our laboratory [28]. The His6-PLIγ and natural saPLIγ were separated under reducing conditions on 12% SDS-PAGE gel, and transferred to different PVDF membranes. Immunodetection was performed by incubating the membranes overnight at 4 °C, stirring, with the PLIγ mAbs at a dilution of 1:500. The reaction was developed using goat anti-mouse peroxidase IgG at a dilution of 1:10,000 and the chemiluminescent substrate Lumi-light (Roche). Images were captured with ChemiDoc XRS^+^ system (Bio-Rad). Parallel Western blot using anti-His6 antibody was conducted to detect His6-PLIγ fusion protein and validate the specificity of PLIγ mAbs.

### PLIγ screening in snake sera

Serum from four venomous snake – including *Bungarus multicinctus, Naja naja, Deinagkistrodon acutus* and *Bungarus fasciatus* – and eight non-venomous snakes – namely *Sinonatrix annularis*, *Zaocys dhumnades*, *Elaphe carinata*, *Dinodon rufozonatum*, *Macropis thodonrudis*, *Elaphe rufodorsata, Elaphe taeniura* and *Achalinus rufescens* – was collected in local market. Western blot analysis of snake sera was performed using the same protocol the abovementioned one. PLIγ mAb was produced by the highest titer hybridoma cell line and purified by protein G affinity chromatography.

### Gene cloning and sequence alignment

In order to validate PLIγ expression in different snakes, gene cloning and DNA sequencing were performed. Due to incomplete samples, snake species did not entirely match those used in PLIγ screening. In brief, we collected fresh liver samples of four venomous snakes – namely *B. multicinctus, N. naja, D. acutus* and *A. halys* – and five non-venomous snakes – including *S. annularis*, *Z. dhumnades*, *E. carinata*, *D. rufozonatum* and *E. rufodorsata.*

Total RNA was extracted from snake liver by using of Trizol reagent (Invitrogen, USA). cDNA was synthesized with SuperScript First Strand Synthesis System (Invitrogen Corp., USA). A pair of degenerate primer was designed based on cDNA sequence of PLIγ as follows: forward: 5’CRCTCATGTAMWTTTGTCACAA3’, reverse primer: 5’TTATTCAGAAGGTGTARTTTTGG3’(where R = A + G; M = A + C; W = A + T). PCR amplification was conducted in 0.2 mL PCR tubes containing 12.5 μL of 2× EasyTaq PCR SuperMix (Transgen, Beijing, China), 1 μL (10 μM) each of forward and reverse primers, 20 ng of cDNA and autoclaved Milli-Q water to make a volume up to 25 μL. Thirty-five cycles of amplification were run as follows: 95 °C denaturation for 30 s, 54 °C annealing for 30 s and 72 °C extension for 30 s. PCR products were sequenced directly with a DYEnamic ET terminator cycle sequencing premix kit (GE Healthcare) on an ABI Prism310 genetic analyzer (Applied Biosystems). Multiple sequence alignment was performed by DNAMAN software (Lynnon Biosoft, USA).

## Results

### Prediction of epitope

**Fig. 1:**
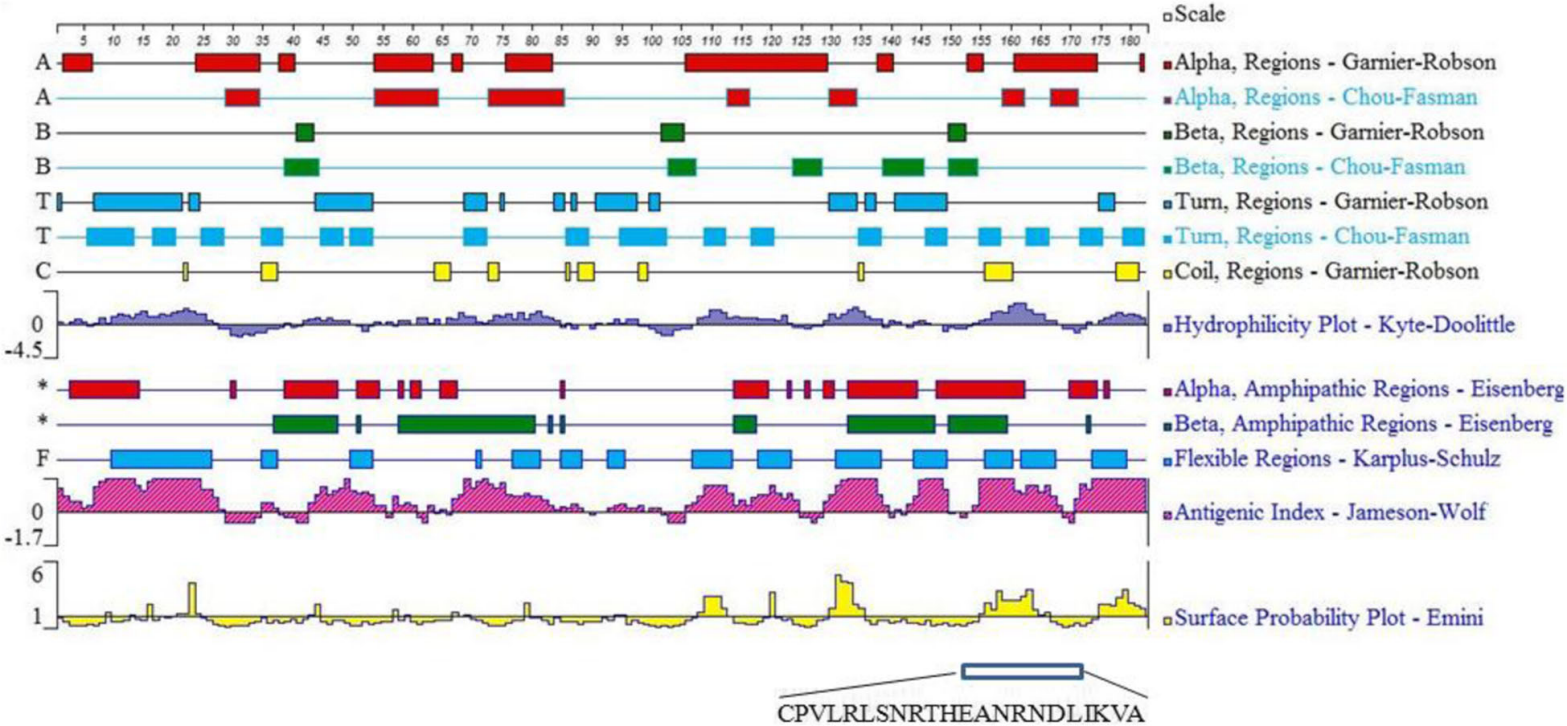
Epitope prediction of PLIγ of *S. annularis* by analyzing secondary structures and other indicators (flexibility, hydrophilicity, antigenicity and surface probability)

The secondary structure, flexible regions, hydrophilicity, surface accessibility and antigenic index were predicted using DNAStar Protean program (Fig. 1). Ideal B cell linear epitopes should be located on the surface of a protein where hydrophilic regions usually exist. Epitopes should contain at least eight amino acids, where the secondary structure should be flexible like β turn and random coil [30]. There are several segments with good hydrophilic and antigenic index in position of 8-27, 68-84, 108-122, 131-136, 151-172 and 176-182, but most of them are poor surface accessible or short than eight amino acids. Based on those rules, a better candidate peptide was selected: ^151^CPVLRLSNRTHEANRNDLIKVA^172^ (Fig. 1).

### Antigen preparation

The peptide (CPVLRLSNRTHEANRNDLIKVA) was called SHE and synthesized by Qiang Yao Biotechnology Company (China). The purity of synthetic SHE was 95% by HPLC assay. In order to increase its immunogenicity, SHE was coupled to KLH (360 kDa) for usage as an immunogen and to BSA (67 kDa) to be used as a coating antigen.

### Immunization and determination of anti-serum titer

**Fig. 2:**
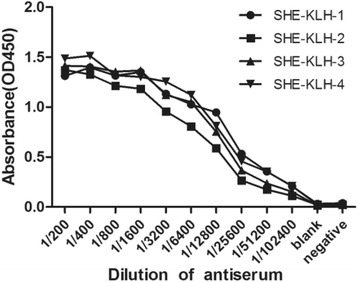
Determination of mouse serum titers by iELISA. SHE-KLH1 ~ SHE-KLH4 represented the four mice that were immunized with SHE-KLH conjugate. The blank received PBS buffer, whereas the negative control received normal mouse serum (1:200)

After four immunization protocols, serum from each mouse was tested for its ability to bind to SHE-BSA by indirect ELISA (iELISA). The serum titers of the immunized Balb/c mice were significantly higher than negative control (injected with PBS buffer only), indicating that SHE-KLH complete antigen induced the production of antibodies. Mouse 1 showed the highest titer and therefore was chosen for further cell fusion with SP2/0 myeloma cells (Fig. 2).

### Cell fusion and selection of positive clones

The fused cells were screened in HAT selective culture medium (only hybridomas from spleen cells and from myeloma cells that successfully fused could grow). At the eighth day of fusion, the hybridoma culture supernatant was detected by indirect ELISA. A total of 44 positive strains were obtained, and the mean positive rate of fusion cells was 5.90%. After the second iELISA screening, 21 positive monoclonal hybridoma cell lines were obtained (Table 1).

**Table 1:** The result of cell fusion and positive hybridoma screening

Plate no.	Fusion rate (%)	Positive rate (%)	Monoclonal hybridomas
1	100% (93/93)	3.23% (3/93)	1C7, 1C11
4	100% (93/93)	5.38% (5/93)	–
5	100% (93/93)	5.38% (5/93)	5D6, 5E9,5E10,5G1
6	100% (93/93)	16.12% (15/93)	6B2,6C12, 6D8, 6E11, 6G11
7	100% (93/93)	1.08% (1/93)	7A10
8	100% (93/93)	8.60% (8/93)	8C7, 8E5,8E12,8G1,8G2
9	100% (93/93)	3.23% (3/93)	9A10,9B8, 9H4
10	100% (93/93)	4.30% (4/93)	10E9
Average	100%	5.90%	

Ten plates (93 wells per plate) were picked and the cell fusion rate were 100%. A total of 44 positive cells were obtained with an average rate of 5.90%. After the second iELISA screening, 21 positive monoclonal hybridoma cell lines were obtained. The nomenclature of positive cell clone was “plate number + row + column”, i.e. 1C7 stands for plate 1, the well of row C (third) and the seventh column of the plate.

### Subtype and immunoblotting analysis of anti-PLIγmAb

Eighteen IgG positive hybridoma cells belonging to three isotypes – IgG1 (5E10, 5G1, 6G11, 7A10); IgG 2a (6C12, 6D8, 6E11, 8E12, 8G1, 8G2, 9H4, 10E9); and IgG2b (1C11, 5D9, 5E9, 6B2, 8C7, 9A10) – were obtained, while the other three were IgA subtype.

**Fig. 3:**
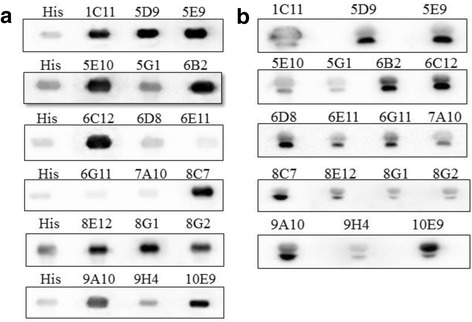
Western blot analysis of the culture supernatant of 18 IgG positive hybridoma clones. **a** Immunoblotting of 18 mAbs to identify recombinant His6-PLIγ. The first lane was blotted with anti-His tagged antibody as positive control, while others were the 18 IgG positive hybridoma culture supernatant. The same position of all lanes indicated that all the 18 mAbs reacted with recombinant *S. annularis* PLIγ. **b** Immunoblotting of the 18 mAbs to identify native *S. annularis* serum*.* The positive bands in all lanes indicated the availability of the 18 mAbs in natural PLIγ. All the natural PLIγ showed two close bands by the immunoblotting, which indicated natural PLIγ is composed of two subunits

A fusion recombinant His6-PLIγ was expressed in prokaryotic system and used for PLIγ mAb validation through regular Western blot method [28]. The immunodetection result is shown in Fig. 3a. His6-PLIγ bands that were detected by His6 antibody or the 18 mAb subtypes migrated to the same position, which indicated that PLIγs were recognized by all the 18 mAbs.

Natural *S. annularis* serum was also used for immunodetection of the 18 mAbs following the same protocol. All the natural PLIγ showed two close bands by immunoblotting, which indicated natural PLIγ is composed of two subunits (Fig. 3b). The result also suggested that the two subunit might be homologous in amino acid sequence. Due to the specificity and sensitivity, we choose 10E9 strain for further amplification of PLIγ mAb preparation.

### Purification of anti-PLIγ mAb

**Fig. 4:**
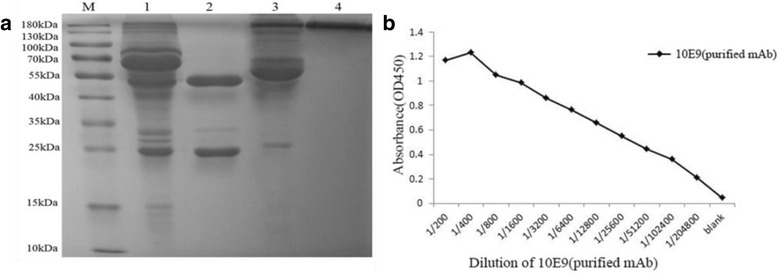
Identification of anti-PLIγ mAb by electrophoresis and titer assay. **a** Reduced and non-reduced electrophoresis of anti-PLIγ mAb on 10% PAGE gel. Samples 1 and 2 were denatured, 3 and 4 were non-denatured. The electrophoretogram indicated the mAb was highly pure. **b** The titer of anti-PLIγ mAb purified from 10E9 strain. M: standard molecular protein marker; lanes 1 and 3: ascites fluid; lanes 2 and 4: purified mAb against PLIγ

The mAbs were purified from ascites fluid by protein G resin affinity chromatography. The peak value of the antibody at 280 nm was 1.65. The electrophoretic graph shows that the protein band results presented the expected size (lane 4, Fig. 4a) under natural PAGE conditions. The peak under reduced PAGE conditions showed two distinct bands, the 54 kDa heavy chain and the 25 kDa light chain (lane 2, Fig. 4a). Indirect ELISA indicated that the titer of purified anti-PLIγ mAb secreted by 10E9 clones was above 1.0 × 10^5^ (Fig. 4b).

### PLIγ screening in snake sera

**Fig. 5:**
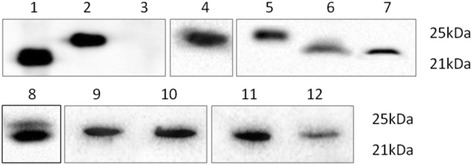
Immunoblot analysis of PLIγ from different snake sera. 1 – *Sinonatrix annularis,* 2 – *Bungarus multicinctus,* 3 – *Bungarus fasciatus*, 4 – *Naja naja*, 5 – *Deinagkistrodon acutus,* 6 – *Macropisthodon rudis,* 7 – *Elaphe carinata, 8 – Zaocys dhumnades, 9 – Elaphe taeniura,* 10 – *Dinodon rufozonatum*, 11 – *Elaphe rufodorsata*, 12 – *Achalinus rufescens*

In order to investigate the application of PLIγmAb, 12 serum samples from different snake species were collected from a local market and underwent immunodetection using anti-PLIγ mAb. Eleven samples showed bands on the film except *Bungarus fasciatus* (lane 3, Fig. 5). Interestingly, the molecular weight of PLIγ in venomous species was usually higher than that of non-venomous snakes. PLIγ levels among these snake sera were also different, there were only 15 μg of in total serum protein loaded in lane 1 (*S. annularis*) and lane 2 (*B. multicinctus*), while other lanes loaded 150 μg serum protein.

### Gene cloning and sequence alignment

**Fig. 6:**
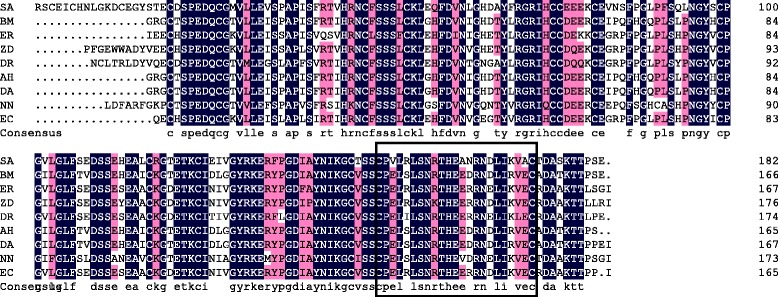
Multiple alignment of PLIγ sequences from different snake species. SA: *S. annularis*; BM: *B. multicinctus;* ER: *E. rufodorsata*; ZD: *Z. dhumnades*; DR: *D. rufozonatum*; AH: *A. halys;* DA: *D. acutus;* NN: *N. naja;* EC: *E. carinata*

The amino acid sequence and alignment of the nine snake PLIγ was shown in Fig. 6. The result indicated a high consistency among different PLIγs (partial CDS), especially in the epitope region (CPVLRLSNRTHEANRNDLIKVA). In addition, the identity of an intraclass PLIγ (i.e. venomous snake only) is higher than interclass. For instance, the amino acids at 155 and 164 of the PLIγ epitope are R/I and R/N in non-venomous snakes PLIγs, whereas they are S and D in venomous snakes.

## Discussion

Snake venom PLA_2_s (svPLA_2_) are closely related to toxicity and comprise an important target for the development of new antivenom drugs. Snake and/or mammals sera are repositories of svPLA_2_ inhibitors (PLIs) due to protective benefits. Immunodetection is an essential technique commonly employed for protein discovery, quantification and investigation. Thus, mAb development of PLIγ is technically significant for antivenom studies.

The classical routine of monoclonal antibody preparation is time-consuming and laborious; the resulted mAbs are generally high specific. Protein-specific antibodies can be generated by immunization of animals with peptides, if the peptide is an effective epitope of the protein. Bioinformatics prediction followed by concrete experimental validation is both economical and effective. For epitope prediction, bioinformatics software can reduce the experimental workload by 95% and increase the efficiency of new epitope location by 10 to 20 folds [31]. In this study, we used DNAStar Protean program to predict epitopes of saPLIγ by comprehensively analyzing many parameters such as hydrophilicity, surface accessibility, antigenic index, secondary structure and flexibility. Finally, we choose ^151^CPVLRLSNRTHEANRNDLIKVA^172^ as a hapten and obtained 18 IgG mAb cell strains. The resulted PLIγ mAb could recognize a broad range of snake sera including venomous and non-venomous snake species, because the epitope peptide is highly homologous among snake PLIγs.

As predicted by Protean program, the saPLIγ has six segments with high antigenic index: 8-27, 68-84, 108-122, 131-136, 151-172 and 176-182. The peptide 8-27aa was good in hydrophilicity, flexibility, antigenicity and had a loose secondary structure (mainly in β-turn), but its surface probability was low, which indicates the epitope may be hidden inside the 3D structure of a protein and unable to act as an antigenic determinant. The peptide 108-122aa was ideal in hydrophilicity, flexibility, antigenicity and surface accessibility, but this segment was a rigid α-helix which might influence its interaction with antibodies. Other segments were too short to be effective epitopes. The best peptide (151-172) generated 18 IgG mAbs that had the ability of binding with recombinant and natural PLIγs.

Epitope prediction and design should also avoid the active amino acid/peptide, otherwise its binding to mAb will block the activity of the protein of interest. The segment ^107^PGLPLSLQNG^116^ in Python’s PLIγ (with N terminal signal peptide) had active sites, as described by Thwin et al. [25]. The saPLIγ also had homologous active peptide as ^87^PGLPFSQLNG^96^, therefore the mAb resulted from ^151^CPVLRLSNRTHEANRNDLIKVA^172^ did not affect the binding of PLIγ to svPLA_2_s and mammal secretory PLA_2_s and is available for coIP investigation (data not shown).

This study showed a wide range distribution of PLIγs in various snake sera. The molecular size of PLIγ in venomous species was generally higher than in non-venomous snakes. Nevertheless, their cDNA sequences were quite identical in length, which indicated that these venomous PLIγs probably undergo more post-translational modification such as glycosylation. The natural PLIγ had been reported to be N-glycosylated on asparagin residues in the sequence Asn-Xaa-Ser/Thr (where Xaa represents any amino acid except proline) [24, 27] at position 178. The modification requires further investigation such as mass spectrum analysis.

Although PLIγs were found in many snake species, their activities were quite different. For example, the saPLIγ have strong anti-hemorrhagic effect and inhibitory activity against venom PLA_2_s from *D .acutus* and *A. halys.* However, the inhibitory effect of PLIγ was weaker against venom PLA_2_s of *B. multicinctus, E. carinata* and *N. naja* [29]. Thus, more efforts should be made on the elaboration of interaction between PLIγs and PLA_2_s, including analysis of active sites, modification and spatial structure difference. The successful preparation of anti-PLIγ antibody is of great impetus for the investigation of the key sequences of natural PLIγs. Effective natural inhibitors have a potential part in the treatment of many diseases involving the PLA_2_ enzyme and also play an important role in bridging the gap between snakebite victims and their successful treatment [23, 32]. It is worth mentioning that PLA_2_ also exists in other animal toxins, such as bee and scorpion venoms [33, 34]. Therefore, inhibition of PLA_2_ is considered a promising lead molecule for a wide spectrum of drugs, including antivenoms and anti-inflammatories.

## Conclusion

A monoclonal antibody against saPLIγ was successfully prepared using epitope prediction with DNAStar Protean program. The resulted anti-PLIγ mAb can be utilized for new PLIγ discovery, quantification and investigation, as well as for the study of interactions between PLIγ and PLA_2_.

## Abbreviations

BSA: Bovine serum albumin
iELISA: Indirect ELISA
KLH: Keyhole limpet hemocyanin
mAb: Monoclonal antibody
PLA_2_: Phospholipase A_2_
PLIγ: Gamma type phospholipase A_2_ inhibitor
SaPLIγ: *Sinonatrix annularis* PLIγ
SIRS: Systematic inflammation response syndrome
svPLA_2_: Snake venom phospholipase A_2_
